# Analyses on the pigment composition of different seed coat colors in adzuki bean

**DOI:** 10.1002/fsn3.2866

**Published:** 2022-04-18

**Authors:** Pu Zhao, Liwei Chu, Kaili Wang, Bo Zhao, Yisong Li, Kai Yang, Ping Wan

**Affiliations:** ^1^ 74684 Key Laboratory of New Technology in Agricultural Application College of Plant Science and Technology Beijing University of Agriculture Beijing China; ^2^ Institute of Modern Agricultural Research Dalian University Liaoning China; ^3^ 74684 Key Laboratory of Urban Agriculture (North) of Ministry of Agriculture College of Bioscience and Resource Environment Beijing University of Agriculture Beijing China

**Keywords:** adzuki bean (*Vigna angularis* L.), anthocyanidin, proanthocyanidin, seed coat color

## Abstract

Seed coat color is an important quality and domestication trait. The adzuki bean has more than a dozen seed coat colors closely associated with the anthocyanin and flavonoid metabolism pathways. In this study, we explored the pigment composition of 10 different seed coat color adzuki beans including red, black mottle on red, black mottle on gray, golden, green, black, ivory, brown, and light brown. The results showed that anthocyanins are the main pigment in adzuki bean seed coat. There were no carotenoid or pelargonidin derivatives in the seed coats of any accessions. Different colors of adzuki bean seed coat have different pigment compositions and the combination of procyanidins and anthocyanins affected seed coat color. The ivory seed coat had an extremely low proanthocyanidin and anthocyanin content. Only the green adzuki bean seed coats contained chlorophyll. Our results explain the pigment composition of the different seed coat colors and the combination of proanthocyanidins and anthocyanins affected seed coat color in adzuki bean. These results can provide a theoretical basis for the study of adzuki bean coloring mechanism.

## Graphical abstract

Anthocyanins are the main pigment in adzuki bean seed coat. Except that the basic contributing factor of green is chlorophyll, different pigment compositions lead to the different colors of adzuki bean seed coat. Proanthocyanidins are not present as copigments in seed coats, and they coexisted with anthocyanidins in the seed coat and have different roles in the coloration. In this study, we explored the pigment composition of 10 different seed coat colour adzuki beans including red, black mottle on red, black mottle on gray, golden, green, black, ivory, brown, and light brown. Different colors of the adzuki bean seed coat have different pigment compositions and the combination of procyanidins and anthocyanins affected seed coat color. Our results explain the pigment composition of the different seed coat colors and the combination of proanthocyanidins and anthocyanins affected seed coat color in adzuki bean.

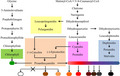

## 1 INTRODUCTION

The adzuki bean (*Vigna angularis*) was domesticated about 12,000 years ago as an important food legume in China (Liu et al., 2013). Almost all the seed coat color of wild adzuki beans (*V*. *angularis var*. *nipponensis*) was black mottle on gray. Artificial domestication and selection have resulted in red, red mottle on white, black mottle on red, black mottle on gray, black, ivory, golden, black mottle on brown, brown, light brown, greenish yellow, greenish white, green, light green, and beige seed coat colors (Li et al., 2017), but red is the main seed coat color of landrace and improved varieties because it is more in line with the traditional food culture (Horiuchi et al., 2015).

Seed coat color can determine the marketability, processability, and nutritional quality of dry beans and has been studied from Mendel's era (Mendel, 1865) to the present day in *Arabidopsis* (Appelhagen et al., 2015), rape (*Brassica napus* L.) (Hong et al., 2017; Rahman et al., 2010), soybean (*Glycine max* L.) (Choung et al., 2001), common bean (*Phaseolus vulgaris* L.) (Beninger & Hosfield, 2003; Beninger et al., 1999), wheat (*Triticum aestivum* L.) (Kohyama et al., 2017), barley (*Hordeum vulgare* L.) (Jia et al., 2020), and many other species. In the common bean, the condensed tannin and anthocyanin contents are correlated with seed coat color (Caldas & Blair, 2009). These metabolites are often detected and identified in seed coat extracts, and researchers believe their levels affect the color, tone, and strength of the seed coat color (Yoshida et al., 1996).

As water‐soluble pigments, flavonoid compounds are widely present in plants (Lepiniec et al., 2006). Proanthocyanidins and anthocyanins are the end products of branched chain flavonoid biosynthesis (Holton & Cornish, 1995; Winkel‐Shirley, 2001). Anthocyanidins determine the color of most tissues and organs in plants, like leaves, flowers, fruits, and seeds, due to the absorption of different wavelengths of visible light (He & Giusti, 2010). It was shown that the presence of anthocyanins plays a significant role in the development of plant adaptative response under abiotic stress effects. For example, the presence of high anthocyanin levels in plants may be an important physiological trait that grants them salinity stress tolerance (Mbarki et al., 2018; Naing & Kim, 2021). Delphinidin, petunidin, malvidin, cyanidin, peonidin, and pelargonidin are the main components of anthocyanidins and they can produce purple, mauve, blue, magenta, crimson, or orange salmon pigments (Bueno et al., 2012; Sytar et al., 2018). In addition, carotenoids (Namitha & Negi, 2010) and chlorophylls are also related to the color formation of plant tissues.

There are few studies on pigment components in the seed coat of adzuki bean. The earliest study was done by Kuroda and Wada (1934). Sasanuma et al. (1966) identified a pigment as 3‐monoglucoside of delphinidin in black red adzuki bean seed coat (Sasanuma et al., 1966). In the red adzuki bean, Yoshida et al. (1996) first reported that the 3‐O‐(β‐D‐glucopyranosyl)‐5‐O‐(β‐D‐glucopyranosyl) cyanidin was the main pigment component in seed coat (Yoshida et al., 1996). Takahama et al. (2013) believe that purplish‐red color of foods is caused by two pigments in adzuki bean (Takahama et al., 2013). Pigments 1 and 2 were isomers, and they were both the products of condensation processes of cyanidin and (+)‐catechin (Takahama et al., 2013). Kawakami et al. (2018) found out two types of polymeric red pigments in adzuki bean, a simple proanthocyanidin and a complicated polyphenol, which was produced from proanthocyanidins (Kawakami et al., 2018). Chu et al. found that Centaurin and delphinidin are the main differential metabolites between red and black adzuki bean seed coat (Chu et al., 2021).

In this study, our results showed that anthocyanidins are the main pigment in adzuki bean seed coat but not carotenoids. The pigment composition and relative quantification of 10 different seed coat color accessions of adzuki bean were assayed by ultraviolet/visible (UV/V) absorption spectroscopy and the ultra‐performance liquid chromatography–tandem mass spectrometry (UPLC–MS/MS), respectively. Anthocyanidins, proanthocyanidins, chlorophylls, and carotenoids were analyzed. The results provide insight into the flavonoid metabolism pathways of adzuki bean seed coat color.

## 2 MATERIALS AND METHODS

### 2.1 Plant materials

**FIGURE 1:**
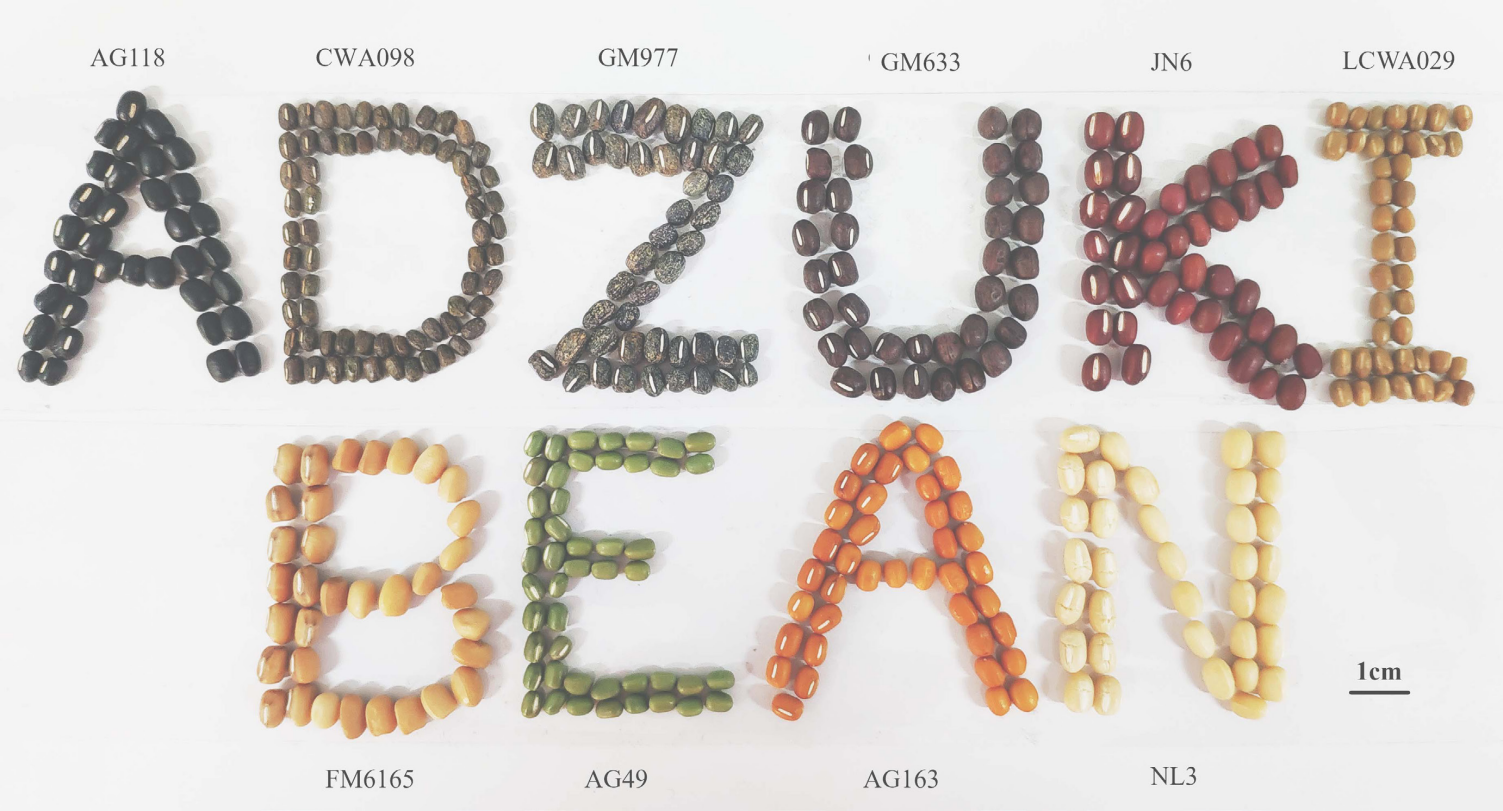
Seed coat colors of 10 adzuki bean accessions

Nine cultigen accessions and one wild accession CWA098 were provided by the Beijing University of Agriculture (BUA) (Table 1 and Figure 1) and were grown at the Experimental Farm of BUA. All of accessions were collected by the BUA. Mutant FM6165, which has a light brown seed coat, was induced with Jingnong6 (JN6) by ethyl methane sulfonate (EMS). Jingnong6 variety was improved and released by BUA. Norin3 is a commercial variety released by Japan. All plant studies were carried out in accordance with relevant institutional, national, or international guidelines and regulation. The adzuki bean used in this study did not involve in endangering or the risk of extinction.

**TABLE 1:** Accessions of different seed coat colors in adzuki bean

No.	Accession	Seed coat color	Type of accession
1	Jingnong6 (JN6)	Red	Improved
2	GM633	Black mottle on red	Landrace
3	GM977	Black mottle on gray	Landrace
4	AG163	Golden	Landrace
5	AG49	Green	Landrace
6	AG118	Black	Landrace
7	Norin3 (NL3)	Ivory	Improved
8	CWA098	Black mottle on brown	Wild
9	LCWA029	Brown	Landrace
10	FM6165	Light brown	Mutant from JN6

### 2.2 Identification of pigments

The pigment identification was conducted according to Li et al. (2020). The seed coat tissues of 10 accessions were ground and flavonoids and carotenoids were extracted using methanol. An equal volume of water and dichloromethane was added to the methanol extract and thoroughly mixed. Finally, the samples were centrifuged at 18000 *g* (revolutions per minute) (5810R, Eppendorf) to separate flavonoids and carotenoids into the supernatant liquid (aqueous) and the denser liquid (no aqueous).

### 2.3 Sample preparation and extraction

Mature seeds of 10 accessions were sprayed with ultrapure water every hour and kept wet for 8 h. When the seed coat started imbibition, the coat was peeled and air‐dried, then freeze‐dried under vacuum. Lyophilized samples were thoroughly ground and extracted with 1 ml of extraction solution for every 100 mg sample, the extraction solution was water:methanol = 3:7, and left to stand for the night at 4°C. Then the overnight extracted solution was centrifuged for 10 min at 10,000 *g*. The CNWBOND Carbon‐GCB SPE Cartridge (ANPEL) was used to absorb the extracts, and the supernatant was filtered by the nylon syringe filter (SCAA‐104, ANPEL) before liquid chromatography–mass spectrometry (LC–MS) analysis.

### 2.4 Preparative and analytical chromatography

The extracts were analyzed using a liquid chromatography–electrospray ionization–tandem mass spectrometry (LC–ESI–MS/MS) system (high‐performance liquid chromatography, HPLC, Shimpack UFLC CBM30A, Shimadzu; mass spectrometry, 4500 QTRAP, Applied Biosystems). The analytical conditions referred to Chen et al.'s methods and were not modified (Chen et al., 2013).

### 2.5 Quality control

The 10 mixed samples were assayed before testing, during testing process, and after testing, respectively, to ensure the stability of the instrument during assay. The relative standard deviation (RSD) is used to test whether the data are qualified. The RSD is the ratio of the standard deviation to the arithmetic mean of the measured result. When the RSD of 85% of the metabolites is less than 0.5%, and 70% of the metabolites is less than 0.3, the quality control is considered qualified.

### 2.6 Identification of chlorophyll and carotenoids by UV/V spectroscopy

Chlorophyll and carotenoid analyses were performed according to previously reported methods, and quantified spectrophotometrically (UV‐1700, Shimadzu) (Gangemi et al., 1987; Poojary & Passamonti, 2015).

### 2.7 Data analyses

The original data were viewed by Analyst software, version 1.6.3, after the mass spectrometry (MS) analyses and to perform qualitative and quantitative analyses. The figure of total ion chromatography (TIC) and multiple reaction monitoring (MRM) extract ion chromatography (XIC) of a mixed sample were applied for quality control (QC). The qualitative and quantitative analyses of the metabolites of samples were based on the local metabolic database (Table S1). All detected metabolites were analyzed using a multipeak mass spectrogram of MRM, and the mass spectral peak of each color represented a metabolite. A triple quadrupole (QQQ) mass spectrometer was used to screen the characteristic ions of each metabolite, and the detector was used to obtain each signal strength of characteristic ions. The resulting files were processed using MultiQuant software. The chromatographic peaks were integrated and corrected. The value of the relative content of metabolites used for study represented the area of the corresponding chromatographic peak. Finally, the above data were exported and saved. The clustering heat map was constructed by the package Pheatmap in R software (version 3.5.1) (Raivo, 2019).

## 3 RESULTS

### 3.1 Pigments' identification of adzuki seed coats

**FIGURE 2:**
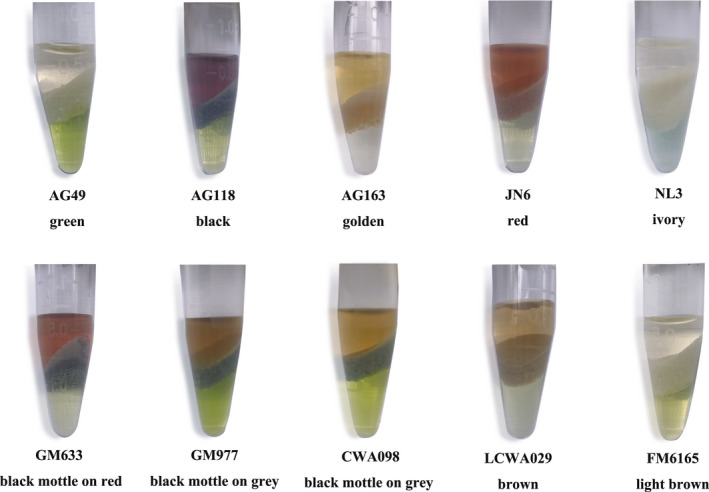
Identification of coloring substances in the adzuki bean seed coats

The pigments of 10 different seed coat color accessions were identified (Table 1). At the same time, it is important to admit that a previous study with anthocyanidins found that the color visual assessment of vegetative organs of plants can be the marker for selection cultivars with high anthocyanin and rutin contents (Sytar et al., 2014). Therefore, in the current study before analysis and during extraction a visual assessment of seeds and extracts was done as well. Flavonoids and anthocyanidins dissolve in the aqueous phase in the upper layer, exhibiting the color consistent with the seed coat. The results showed that the different colors of seed coat in adzuki bean were mainly determined by anthocyanidins and not carotenoids (Figure 2).

### 3.2 Quality control

We mixed 10 samples and assayed the mixed sample three times before, during, and after testing, respectively, to ensure the stability of the instrument during assay. The TIC results of the QC samples are shown in Figure S1. The quality control data have been submitted to Table S2. The curves, retention time, and peak strength were all consistent. These results suggest that the signal was stable when MS was used to analyze the sample at different times.

### 3.3 Qualitative and relative quantitative analyses of metabolites

There are eight different known anthocyanidin metabolites and four different known proanthocyanidin metabolites that were detected (Table 2). The anthocyanins were cyanidin (Cy), cyanidin‐3‐O‐glucoside (Cy‐glu), delphinidin‐3‐O‐glucoside (Dp‐glu), malvidin‐3,5‐diglucoside (Mv‐dig), malvidin‐3‐O‐galactoside (Mv‐gal), malvidin‐3‐O‐glucoside (Mv‐glu), peonidin‐O‐hexoside (Pe‐hex), and petunidin‐3‐O‐glucoside (Pt‐glu), and the proanthocyanidins were procyanidin A1, procyanidin A2, procyanidin B2, and procyanidin B3.

**TABLE 2:** Anthocyanins and proanthocyanidins detected among 10 adzuki bean seed coat samples

Index	Q1 (Da)	Q3 (Da)	Rt (min)	Molecular weight (Da)	Ionization model	Compounds	Class
1	287	213	3.37	287.2	Protonated	Cyanidin	Anthocyanins
2	449.1	287.3	2.55	449.1	Protonated	Cyanidin‐3‐O‐glucoside	Anthocyanins
3	463.1	301	3.02	463.1	Protonated	Peonidin‐O‐hexoside	Anthocyanins
4	465.1	303	2.41	465.1	Protonated	Delphinidin‐3‐O‐glucoside	Anthocyanins
5	655.2	331.1	2.52	655.2	Protonated	Malvidin‐3,5‐diglucoside	Anthocyanins
6	493	331	2.77	493	Protonated	Malvidin‐3‐O‐galactoside	Anthocyanins
7	493.2	331	2.84	493.2	Protonated	Malvidin‐3‐O‐glucoside	Anthocyanins
8	479	317	2.61	479	Protonated	Petunidin‐3‐O‐glucoside	Anthocyanins
9	577	287	3.62	576.1	[M + H]+	Procyanidin A1	Proanthocyanidins
10	575	285	3.89	576.1	[M − H]−	Procyanidin A2	Proanthocyanidins
11	579.1	427.1	3.04	578.1	[M + H]+	Procyanidin B2	Proanthocyanidins
12	577.1	407.1	2.83	578.1	[M − H]−	Procyanidin B3	Proanthocyanidins

### 3.4 Cluster heat map

**FIGURE 3:**
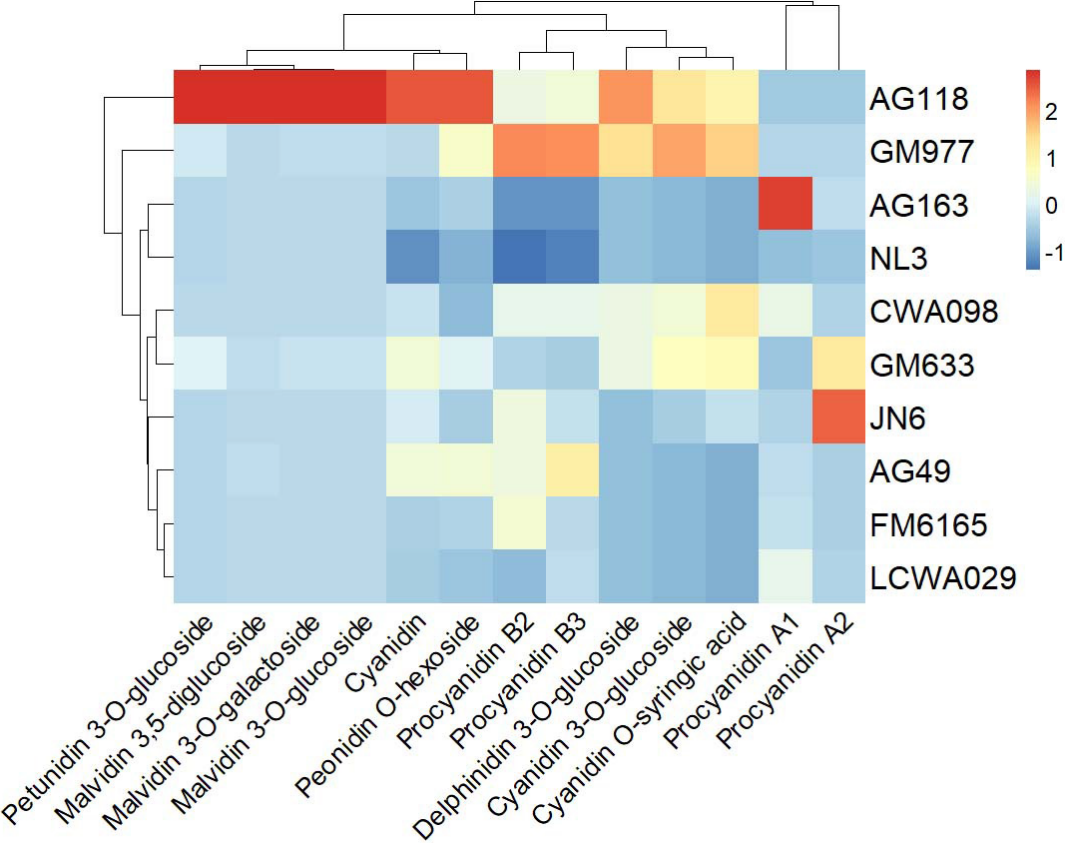
Clustering heat map of the relative metabolite content in adzuki bean seed coats

In cluster heat map (Figure 3) analyses, the brown seed coat LCWA029 and light brown seed coat FM6165, a mutant from the red seed coat JN6, were clustered together. GM633 with black mottle on red seed coat and CWA098, a wild adzuki bean with black mottle on brown seed coat, were clustered together, probably because similar metabolites led to the same black mottle phenotype. There were few pigment types leading to ivory and golden seed coats, and the content of most components was very low. Therefore, they were clustered together. The green seed coat AG49 was clustered with the brown LCWA029 and light brown seed coat FM6165, and they contained similar metabolites. But chlorophyll content was not included in this cluster analysis. The red seed coat JN6 was spotted independently in a branch. Both the black AG118 and black mottle on gray GM977 seed coats occupied together a branch, and their pigment composition of seed coats was relatively similar.

The cluster analyses and heat map indicated that there were significant differences in pigment components in different seed coats, and semblable seed coats have certain similarities in pigment components.

### 3.5 Pigment components of adzuki bean seed coats

There was a complex array of pigment components and there were differences in their contents among the different seed coat colors.

The ivory seed coat Norin3 contained very small amounts of Cy‐glu and Pt‐glu (Table 3). The anthocyanidin content in the brown LCWA029 and light brown FM6165 seed coats was low, while the procyanidin A1 content was high. Procyanidin B3 was the predominant pigment (Table 3). The procyanidin A1 and procyanidin B2 contents were different between FM6165 and LCWA029 (Table S3).

**TABLE 3:** Relative amounts of anthocyanidin and proanthocyanidin contents in 10 adzuki bean seed coat accessions

Compounds (10^5^)	AG118	CWA098	GM977	GM633	JN6	LCWA029	FM6165	AG49	AG163	NL3
Cyanidin	0.80	0.22	0.20	0.36	0.26	0.17	0.18	0.35	0.15	0.03
Cyanidin‐3‐O‐glucoside	277.00	165.00	368.00	199.00	31.90	0.80	0.88	1.07	1.41	1.13
Peonidin‐O‐hexoside	1.62	0.10	0.71	0.47	0.20	0.16	0.24	0.64	0.23	0.05
Delphinidin‐3‐O‐glucoside	60.30	20.80	45.10	20.70	0.14	0.08	0.08	0.17	0.17	0.07
Malvidin‐3,5‐diglucoside	242.00	0.09	0.16	1.56	0.09	0.01	0.02	1.07	0.02	0.07
Malvidin‐3‐O‐galactoside	82.60	0.00	0.57	2.72	0.00	0.00	0.00	0.17	0.00	0.00
Malvidin‐3‐O‐glucoside	159.00	0.25	0.97	5.27	0.00	0.00	0.00	0.22	0.00	0.00
Petunidin‐3‐O‐glucoside	704.00	9.98	65.00	88.30	0.54	0.39	0.36	1.76	0.29	1.62
Procyanidin A1	0.43	3.71	1.16	0.38	0.96	3.64	1.82	1.64	14.40	0.01
Procyanidin A2	0.35	1.18	1.28	11.50	19.30	1.12	0.70	0.73	1.83	0.00
Procyanidin B2	7.07	6.64	14.80	4.28	7.27	2.91	8.01	7.25	1.31	0.01
Procyanidin B3	158.00	135.00	316.00	73.70	96.70	90.50	88.00	220.00	17.30	0.00

The red JN6 had a higher procyanidin A2 content than the other colors and also had high levels of procyanidin B3 and Cy‐glu (Table 3). The light brown seed coat FM6165 was a mutant from JN6 that was induced by EMS. The red seed coat JN6 contained Cy‐glu and procyanidin A2, and Cy‐glu content in JN6 was significantly higher than in FM6165 (Table S4). These two metabolites were responsible for the differences leading to red or light brown seed coat coloration, particularly Cy‐glu, which is a known red pigment 16. The FM6165 mutant deprived red seed coat phenotype of JN6.

The levels of procyanidin A2 between the red JN6 and black mottle on red GM633 showed no significant differences, but the levels in both types were significantly higher than those in other color varieties (Table S5).

Procyanidin A1 and procyanidin B3 were detected in the golden seed coat AG163. The procyanidin A1 content was higher than that in the other seed coat colors. The procyanidin B3 content was significantly lower than that in other seed coat colors, except the ivory Norin3 seed coat (Table S6). Procyanidin A1 was involved in the formation of the golden seed coat coloration. Carotenoids were not detected (Figure S2), indicating that there were no carotenoids or pelargonidin in adzuki bean.

The pigment components of black or black mottled seed coats in adzuki bean are complex, and they tend to have high contents of Pt‐glu, Cy‐glu, and Dp‐glu.

The black seed coats accumulated higher contents of petunidin and malvidin derivatives than other colored seeds. These derivatives are Pt‐glu, Mv‐dig, Mv‐gal, Mv‐glu, and Dp‐glu. Also, black seed coats had high levels of Cy‐glu and procyanidin B3 but were not higher than the rest of the colored seed coats.

There were very significant differences in the Pt‐glu, Dp‐glu, and Cy‐glu contents between the black mottle on red GM633 and red JN6 seed coats, and also between the black mottle on brown CWA098 and brown LCWA029 seed coats. We did not find significant differences in the proanthocyanidin contents between the black mottle and no black mottle‐colored seed coats (Table S5). The main components of the black mottled seed coat were Pt‐glu, Dp‐glu, and Cy‐glu.

The green coat had a similar composition to those of the brown and light brown coats and clustered with them (Figure 3). The procyanidin B3 content was high (Table 3), but only the green coat contained chlorophyll (Figure S2) which did not appear in the cluster heat map. In particular, the green coat color may be mainly due to the presence of chlorophyll, as suggested by previous studies (Lee et al., 2009).

### 3.6 Biosynthetic pathway model of pigment components in adzuki bean seed coats

**FIGURE 4:**
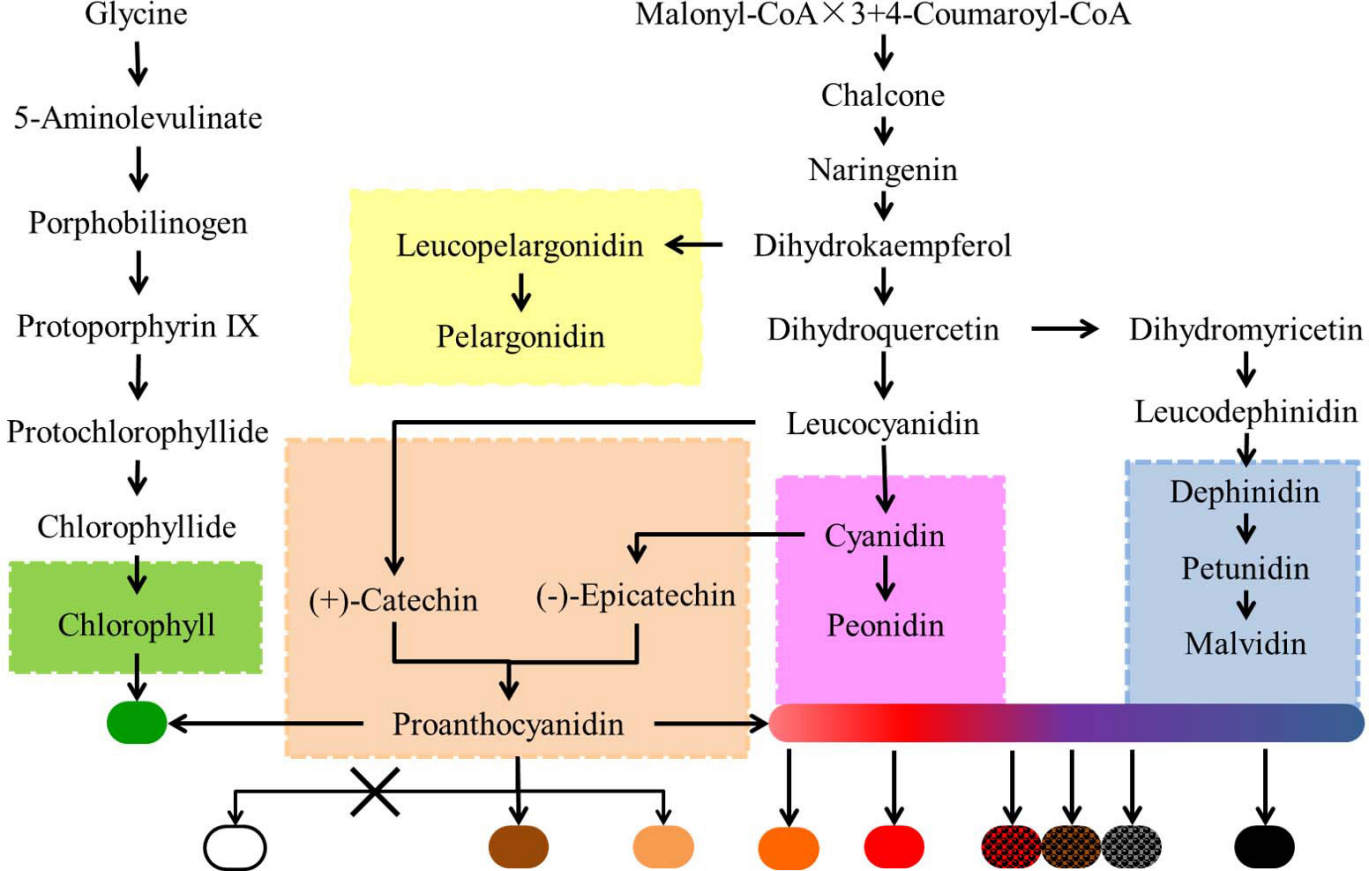
A biosynthetic pathway model of the pigment components in 10 adzuki bean seed coat colors

Based on the above results, we predicted a biosynthetic pathway model of pigment components of 10 seed coat colors in adzuki bean (Figure 4) (Lepiniec et al., 2006).

The ivory seed coat exhibited a lack of prothoancyanidins and has no accumulated pigment. Brown, light brown, and brown adzuki bean seed coats contained higher levels of procyanidin A1. There was a low content of the Cy monomer in all varieties, while there were high levels of Cy‐glu in the seed coats of black, black mottle on gray, black mottle on red, and red adzuki beans. The Dp‐glu and Pt‐glu contents were high in the black mottle and black seed coat of adzuki beans. The main compounds for the black seed coat were Cy‐glu, Dp‐glu, Pt‐glu, malvidin derivatives, and procyanidin B3. Cy‐glu, Pt‐glu, and Dp‐glu contributed to the black mottle formation in adzuki beans. The procyanidin B3 content was high in all varieties, except the ivory adzuki bean, and the procyanidin A2 content was high only in the red seed coat. The procyanidin A1 content was only high in the golden adzuki beans. Green adzuki beans contained chlorophyll and had high accumulations of procyanidin B3.

## 4 DISCUSSION

Free anthocyanidins have no obvious color in weakly acidic plant vacuoles and need to be colored by copigmentation. Cai et al. (1990) reported that prothoancyanidins displayed very small copigmentation effects (Cai et al., 1990). In a previous study of common bean, there were some correlations between the tannin concentration and seed coat color, but this correlation was weak (Caldas & Blair, 2009). Proanthocyanidins are the main pigments in the brown seed coat of soybean (Zabala & Vodkin, 2003), common bean (Beninger & Hosfield, 2003), and *Arabidopsis thaliana* (Abrahams et al., 2002; Koornneef, 1990). A reduction in the accumulation of proanthocyanidins was shown to diminish the brown seed coloration in *Arabidopsis* (Appelhagen et al., 2015). In *Arabidopsis*, seed coats with a low proanthocyanidin content are transparent and their color varies from buff to pale brown (Abrahams et al., 2002; Koornneef, 1990). The situation is similar in soybean, where proanthocyanidins are also found in the brown and buff soybean seed coats (Zabala & Vodkin, 2003). In this study, the proanthocyanidin content of the ivory Norin3 seed coat was extremely low, but other seed coat color accessions contained a large amount of proanthocyanidins. Proanthocyanidins were involved in the formation of golden seed coat coloration and the seed coat color difference between red and light brown. Our results indicated that proanthocyanidins are not present as copigments in seed coats, and they coexisted with anthocyanidins in the seed coat and have different roles in the coloration.

In soybean, the black seed coat of soybeans mainly contains cyanidin, delphinidin, and petunidin (Buzzell et al., 1987; Lee et al., 2009, 2016; Todd & Vodkin, 1993). Choung et al. (2001) found that Cy‐glu was the most common metabolite in the black seed coat (Choung et al., 2001). Delphinidin is accumulated in large quantities in blue grain wheat and barley (Jia et al., 2020). Dp‐glu was isolated from the black red adzuki bean (Cho et al., 2013). We suggest that malvidin derivatives and Pt‐glu are necessary metabolites for producing the black seed coat in adzuki beans, and Cy‐glu and Dp‐glu also participate. In addition, Cy‐glu, Dp‐glu, and Pt‐glu are involved in the formation of black mottle (Figure 4). The black seed coat might be compounded by several pigments in adzuki bean. In soybean, the difference of pigment accumulation area in seed coat was caused by gene expression specificity in time and space (Cho et al., 2017). It is also possible that the black mottle is mainly due to the different area on seed coat distribution of black pigment.

The wild adzuki bean has a small seed and a black mottle on gray seed coat color, which provides effective protection. Most of the adzuki bean cultigens have red seed coats, although some landraces have other colors. But wild soybean has a black or green seed coat color to avoid predation, while the most cultivated soybean has a yellow seed coat (Wang et al., 2018). Wild plant seed color has evolved to be a close match to the color of the prevailing natural environment, enabling seeds to avoid detection by seed predators (Porter, 2013). Results in this study indicated that pigments in adzuki bean seed coat of different colors might be related to domestication but the mechanism of seed coat color change during adzuki bean domestication needs further study. Anthocyanidins and proanthocyanidins are the main pigments in adzuki bean seed coat, which have anti‐oxidation and anticancer properties. In this study, we analyzed the pigment compositions of different seed coat colors in adzuki beans, and predicted the biosynthetic pathway of pigment components. The results provide new insights into the pigment metabolic mechanism of seed coats in adzuki beans.

## CONFLICT OF INTEREST

The authors declare no competing financial interests.

## Supporting information

Figure S1

Figure S2

Table S1‐S6

## Data Availability

The authors confirm that the data supporting the findings of this study are available within the article [and/or] its supplementary materials.
