# Supplementary figures and images for: Analyses on the pigment composition of different seed coat colors in adzuki bean
Source: Food Sci Nutr. 2022 Apr 18;10(8):2611–9. doi: 10.1002/fsn3.2866 (PMC9361439; doi:10.1002/fsn3.2866)

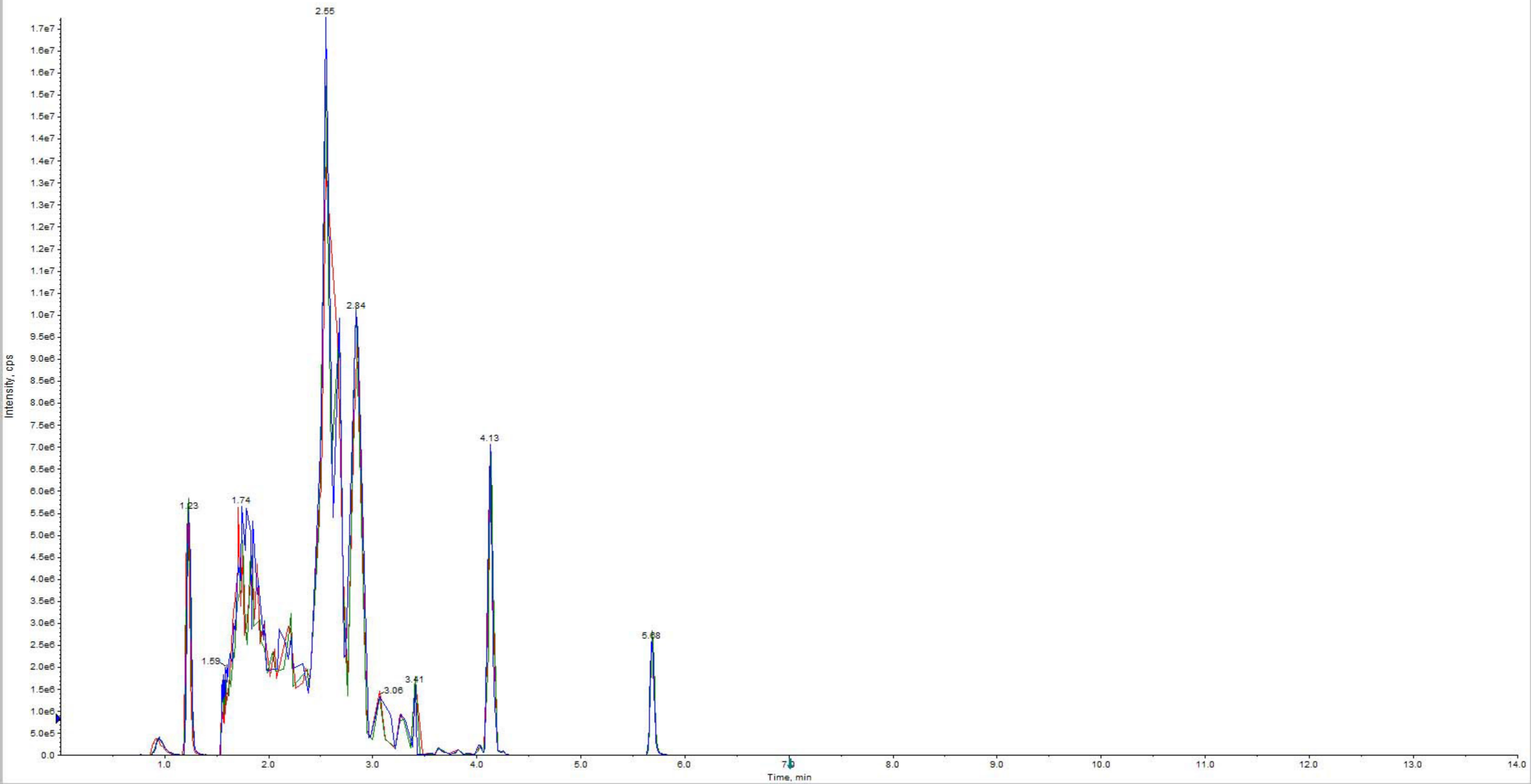

Supplement: Supplementary file 1 — Figure S1 [file FSN3-10-2611-s003.pdf]

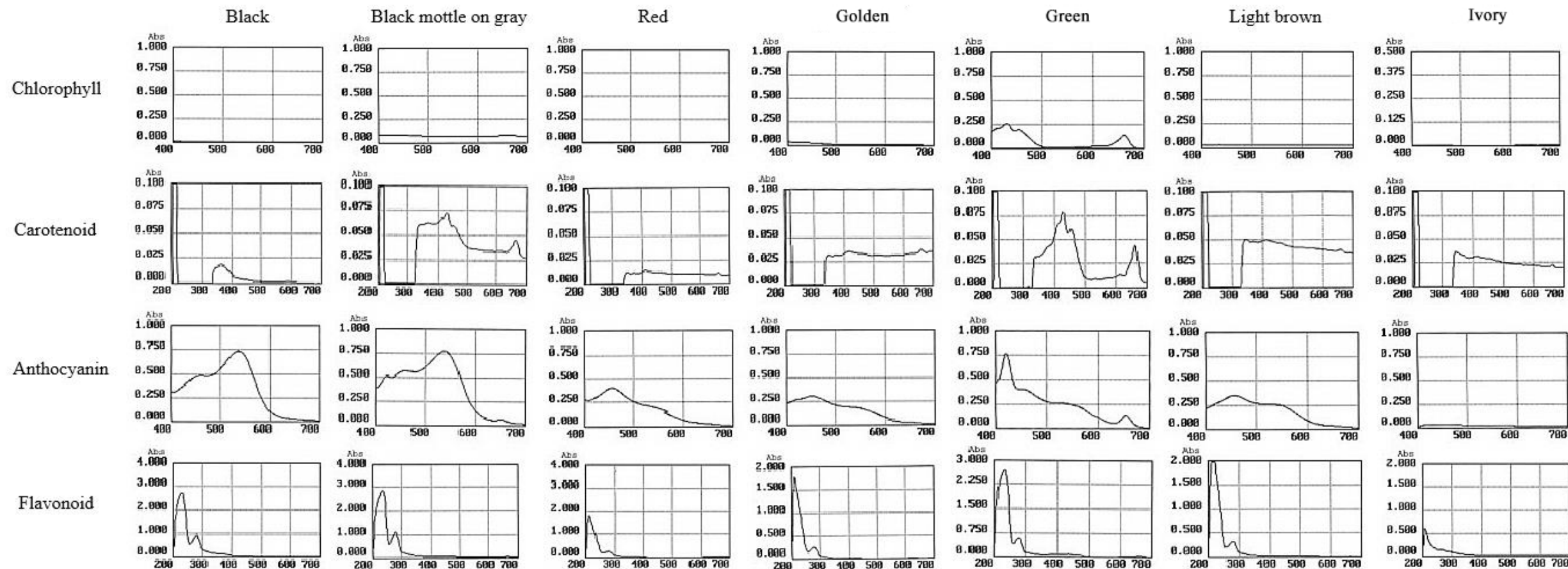

Supplement: Supplementary file 2 — Figure S2 [file FSN3-10-2611-s001.pdf]
