# Supplementary material for: Analyses on the pigment composition of different seed coat colors in adzuki bean
Source: Food Sci Nutr. 2022 Apr 18;10(8):2611–9. doi: 10.1002/fsn3.2866 (PMC9361439; doi:10.1002/fsn3.2866)
Supplement: Supplementary file 3 — Table S1‐S6 [file FSN3-10-2611-s002.docx]

Table S1 46 metabolites in the database

| Compounds | Classes |
| --- | --- |
| Delphinidin 3,5-O-diglucoside | Delphinidin |
| Delphinidin 3-O-galactoside | Delphinidin |
| Delphinidin 3-O-glucoside | Delphinidin |
| Delphinidin 3-O-arabinoside | Delphinidin |
| Delphinidin 3-O-rutinoside | Delphinidin |
| Delphinidin 3-O-(6''-O-malonyl)-beta-D-glucoside | Delphinidin |
| Cyanidin 3,5-O-diglucoside | Cyanidin |
| Cyanidin 3-O-galactoside | Cyanidin |
| Cyanidin 3-O-glucoside | Cyanidin |
| Cyanidin 3-O-arabinoside | Cyanidin |
| Cyanidin 3-O-rutinoside | Cyanidin |
| Cyanidin 3-O-(6-O-malonyl-beta-D-glucoside) | Cyanidin |
| Petunidin 3,5-diglucoside | Petunidin |
| Petunidin 3-O-galactoside | Petunidin |
| Petunidin 3-O-glucoside | Petunidin |
| Petunidin 3-O-arabinoside | Petunidin |
| Petunidin 3-O-rutinoside | Petunidin |
| Petunidin 3-O-(6-O-malonyl-beta-D-glucoside) | Petunidin |
| Pelargonidin 3,5-O-diglucoside | Pelargonidin |
| Pelargonidin 3-O-galactoside | Pelargonidin |
| Pelargonidin 3-O-glucoside | Pelargonidin |
| Pelargonidin 3-O-arabinoside | Pelargonidin |
| Pelargonidin 3-O-rutinoside | Pelargonidin |
| Pelargonidin 3-O-(6-O-malonyl-beta-D-glucoside) | Pelargonidin |
| Peonidin 3,5-O-diglucoside | Peonidin |
| Peonidin 3-O-galactoside | Peonidin |
| Peonidin 3-O-glucoside | Peonidin |
| Peonidin 3-O-arabinoside | Peonidin |
| Peonidin 3-O-rutinoside | Peonidin |
| Peonidin 3-O-(6-O-malonyl-beta-D-glucoside) | Peonidin |
| Malvidin 3,5-diglucoside | Malvidin |
| Malvidin 3-O-galactoside | Malvidin |
| Malvidin 3-O-glucoside | Malvidin |
| Malvidin 3-O-arabinoside | Malvidin |
| Malvidin 3-O-rutinoside | Malvidin |
| Malvidin 3-O-(6-O-malonyl-beta-D-glucoside) | Malvidin |
| Aromadendrin (Dihydrokaempferol) | Flavone |
| Chalcone | Flavone |
| Dihydromyricetin | Flavone |
| Quercetin3-O-glucoside | Flavone |
| ProcyanidinA2 | Proanthocyanidin |
| ProcyanidinA1 | Proanthocyanidin |
| ProcyanidinB2 | Proanthocyanidin |
| ProcyanidinB3 | Proanthocyanidin |
| ProcyanidinB1 | Proanthocyanidin |
| ProcyanidinC1 | Proanthocyanidin |

Table S2 The relative standard deviation (RSD) of quality control

| Compounds (10^5^) | mix01 | mix02 | mix03 | SD | Mean | RSD |
| --- | --- | --- | --- | --- | --- | --- |
| Cyanidin | 0.27 | 0.26 | 0.26 | 0.01 | 0.26 | 0.04 |
| Cyanidin 3-O-glucoside | 178.00 | 153.00 | 155.00 | 13.89 | 162.00 | 0.09 |
| Delphinidin 3-O-glucoside | 24.50 | 21.50 | 20.30 | 2.16 | 22.10 | 0.10 |
| Malvidin 3,5-diglucoside | 64.60 | 56.30 | 58.70 | 4.27 | 59.87 | 0.07 |
| Malvidin 3-O-galactoside | 13.50 | 12.70 | 13.50 | 0.46 | 13.23 | 0.03 |
| Malvidin 3-O-glucoside | 21.80 | 21.80 | 22.20 | 0.23 | 21.93 | 0.01 |
| Peonidin O-hexoside | 0.69 | 0.59 | 0.48 | 0.11 | 0.59 | 0.18 |
| Petunidin 3-O-glucoside | 287.00 | 244.00 | 249.00 | 23.52 | 260.00 | 0.09 |
| Procyanidin A1 | 1.03 | 1.36 | 1.21 | 0.17 | 1.20 | 0.14 |
| Procyanidin A2 | 4.33 | 4.16 | 4.21 | 0.09 | 4.23 | 0.02 |
| Procyanidin B2 | 9.34 | 9.22 | 9.03 | 0.16 | 9.20 | 0.02 |
| Procyanidin B3 | 174.00 | 160.00 | 159.00 | 8.39 | 164.33 | 0.05 |

Table S3 Differences in the procyanidin content between LCWA029 and FM6165

| Compounds | LCWA029 | FM6165 | Fold Change | Log_2_（FC） |
| --- | --- | --- | --- | --- |
| Procyanidin A1 (10^5^) | 3.64 | 1.82 | 0.50 | -1.00 |
| Procyanidin A2 (10^5^) | 1.12 | 0.70 | 0.63 | -0.68 |
| Procyanidin B2 (10^5^) | 2.91 | 8.01 | 2.75 | 1.46 |
| Procyanidin B3 (10^5^) | 90.50 | 88.00 | 0.97 | -0.04 |

Table S4 Differences in the metabolite contents between Jingnong6 and FM6165

| Compounds | JN6 | FM6165 | Fold Change | Log_2_（FC） |
| --- | --- | --- | --- | --- |
| Cyanidin 3-O-glucoside (10^5^) | 31.9 | 0.88 | 36.25 | 5.18 |
| Procyanidin A2 (10^5^) | 1.93 | 0.70 | 2.76 | 1.46 |

Table S5 Analyses of the significant differences in the procyanidin A2 content between Jingnong6 and the other accessions

| Compounds | AG118 | CWA098 | GM977 | GM633 | JN6 | LCWA029 | FM6165 | AG49 | AG163 | NL3 |
| --- | --- | --- | --- | --- | --- | --- | --- | --- | --- | --- |
| Procyanidin A2 (10^5^) | 0.35 | 1.18 | 1.28 | 11.50 | 19.30 | 1.12 | 0.70 | 0.73 | 1.83 | 0.00 |
| Fold Change | 55.14 | 16.36 | 15.08 | 1.68 | 1.00 | 17.23 | 27.57 | 26.44 | 10.55 | NA |
| Log_2_（FC） | 5.79 | 4.03 | 3.91 | 0.75 | 0.00 | 4.11 | 4.79 | 4.72 | 3.40 | NA |

Table S6 Analyses of the significant differences in the procyanidin A1 and procyanidin B3 contents between AG163 and the other accessions

| Compounds | AG118 | CWA098 | GM977 | GM633 | JN6 | LCWA029 | FM6165 | AG49 | AG163 | NL3 |
| --- | --- | --- | --- | --- | --- | --- | --- | --- | --- | --- |
| Procyanidin A1（10^5^） | 0.43 | 3.71 | 1.16 | 0.38 | 0.96 | 3.64 | 1.82 | 1.64 | 14.4 | 0.01 |
| Fold Change | 33.49 | 3.88 | 12.41 | 37.89 | 15.00 | 3.96 | 7.91 | 8.78 | 1.00 | 1440.00 |
| Log_2_（FC） | 5.07 | 1.96 | 3.63 | 5.24 | 3.91 | 1.98 | 2.98 | 3.13 | 0.00 | 10.49 |
| Procyanidin B3（10^5^） | 158 | 135 | 316 | 737 | 96.7 | 90.5 | 88 | 220 | 17.3 | 0.00 |
| Fold Change | 0.11 | 0.13 | 0.05 | 0.23 | 0.18 | 0.19 | 0.20 | 0.08 | 1.00 | NA |
| Log_2_（FC） | -3.19 | -2.96 | -4.19 | -2.09 | -2.48 | -2.39 | -2.35 | -3.67 | 0.00 | NA |
